# Perceptions and practices regarding light sedation in mechanically ventilated patients: a survey on the attitudes of Brazilian critical care physicians

**DOI:** 10.5935/0103-507X.20220278-en

**Published:** 2022

**Authors:** Vicente Cés de Souza-Dantas, Lilian Maria Sobreira Tanaka, Rodrigo Bernardo Serafim, Jorge Ibrain Figueira Salluh

**Affiliations:** 1 Postgradute Program in Translational Medicine, Instituto D’Or de Pesquisa e Ensino - Rio de Janeiro (RJ), Brazil.; 2 Hospital Copa D’Or - Rio de Janeiro (RJ), Brazil.; 3 Intensive Care Unit, Hospital Copa D’Or - Rio de Janeiro (RJ), Brazil.; 4 Department of Intensive Care, Instituto D’Or de Pesquisa e Ensino - Rio de Janeiro (RJ), Brazil.

**Keywords:** Conscious sedation, Intensive care units, Respiration, artificial, Health knowledge, attitudes, practice, Surveys and questionnaires

## Abstract

**Objective:**

To characterize the knowledge and perceived attitudes toward pharmacologic interventions for light sedation in mechanically ventilated patients and to understand the current gaps comparing current practice with the recommendations of the Clinical Practice Guidelines for the Prevention and Management of Pain, Agitation/Sedation, *Delirium*, Immobility, and Sleep Disruption in Adult Patients in the Intensive Care Unit.

**Methods:**

This was a cross-sectional cohort study based on the application of an electronic questionnaire focused on sedation practices.

**Results:**

A total of 303 critical care physicians provided responses to the survey. Most respondents reported routine use of a structured sedation scale (281; 92.6%). Almost half of the respondents reported performing daily interruptions of sedation (147; 48.4%), and the same percentage of participants (48.0%) agreed that patients are often over sedated. During the COVID-19 pandemic, participants reported that patients had a higher chance of receiving midazolam compared to before the pandemic (178; 58.8% *versus* 106; 34.0%; p = 0.05), and heavy sedation was more common during the COVID-19 pandemic (241; 79.4% *versus* 148; 49.0%; p = 0.01).

**Conclusion:**

This survey provides valuable data on the perceived attitudes of Brazilian intensive care physicians regarding sedation. Although daily interruption of sedation was a well-known concept and sedation scales were often used by the respondents, insufficient effort was put into frequent monitoring, use of protocols and systematic implementation of sedation strategies. Despite the perception of the benefits linked with light sedation, there is a need to identify improvement targets to propose educational strategies to improve current practices.

## INTRODUCTION

Sedatives are routinely used in patients in the intensive care units (ICU) to provide comfort, relieve anxiety and reduce stress, improving tolerance to invasive procedures as well as ensuring synchrony to invasive mechanical ventilation (MV).^(1)^ Current evidence supports the use of light sedation levels to achieve the abovementioned goals, with only a minority of patients requiring continuous deep sedation. The optimal sedation level varies widely across patients depending on their clinical condition and the treatment needed.^(2)^ Therefore, sedation level assessment and monitoring should be routinely performed in ICUs.^(2)^

The Clinical Practice Guidelines for the Prevention and Management of Pain, Agitation/Sedation, *Delirium*, Immobility, and Sleep Disruption in Adult Patients in the ICU (PADIS guidelines) concluded that light sedation in patients in the ICU was significantly associated with a shorter extubation time and reduced tracheostomy rate.^(1)^ However, in recent decades, substantial evidence has demonstrated the detrimental impact of poor sedation practices on the outcomes of ICU patients.^(3)^

The ideal sedation strategy for critically ill patients should address pain, sedation, and anxiety; have favorable kinetics and clinical effects; be easily titrated and monitored; have a tolerable side effect profile; and be affordable.^(1,3)^ In recent years, several surveys have been published on the practice of sedation aiming to reflect current practices and their corresponding changes considering new evidence.^(4-6)^

Despite all of the available data, currently employed sedation practices are still heterogeneous regarding adherence to current recommendations.^(1)^ Moreover, the COVID-19 pandemic has substantially changed general care practices in ICUs, including sedation and analgesia strategies for MV patients.^(5)^

We conducted a survey of Brazilian ICU physicians aiming to characterize the knowledge and perceived attitudes toward pharmacologic interventions for sedation and to understand the current gaps comparing current practice with the recommendations of the PADIS guidelines.

## METHODS

### Survey development and administration

We conducted a nonsystematic Medlin® e search of the literature on “sedation,” “light sedation”, “mechanical ventilation,” and “ICU” to identify the most relevant evidence on sedation practices. We subsequently summarized the current evidence and used it to develop the questionnaire.

This resulted in a 2-part questionnaire that evaluated the respondents and their related ICU characteristics (10 questions) and sedation practices (8 questions). The self-administered questionnaire (Supplementary material) was constructed on an electronic web-based system (www.surveymonkey.com).

The survey did not contain data that could identify the respondents. The Institutional Review Board approved the study and waived the need for informed consent.

From August 15 to September 15, 2021, an invitation to complete the survey was disseminated through social media and sent by email to a convenience sample of ICU physicians using the mailing list of *Instituto D’Or de Pesquisa e Ensino*. Respondents were instructed to complete the survey only one time.

### Data and statistical analysis

The survey results were exported into a Microsoft Excel 16.0 (Microsoft^®^, New Mexico, United States) template and analyzed using the Statistical Package for the Social Sciences 23.0 (SPSS, IBM^®^, New York, United States).

Standard descriptive statistics were used as appropriate. Variables were reported as numbers (percentages). As the number of respondents varied across the questions, the proportions displayed in the results section and tables are not constant. Fisher’s exact test was used for the comparison of the variables. A 2-sided P value of less than.05 was considered significant.

## RESULTS

### Demographics

A total of 303 critical care physicians provided responses to the survey. The main respondents´ demographics and ICU characteristics are depicted in table 1. Respondents represented from all geographic regions of the country. A total of 98% of respondents provided complete responses and were included in the analysis.

**Table 1:** The main respondents’ demographics and intensive care unit characteristics

Geographic regions	
Midwest	14 (4)
Northeast	27 (9)
North	2 (1)
Southeast	239 (79)
South	21 (7)
Years of ICU practice	
1 - 5	94 (31)
5 - 10	79 (26)
> 10	130 (43)
Main practice setting	
Academic medical center	142 (43)
Nonacademic medical center	161 (57)
Public hospital	106 (35)
Private hospital	197 (65)
ICU beds	
1 - 10	109 (36)
11 - 20	106 (35)
> 20	88 (29)
Daily multidisciplinary rounds in the ICU	
Have daily rounds	236 (78)
No daily rounds	67 (22)

ICU - intensive care unit. Results expressed as n (%).

Overall, 125 (40.8%) respondents were board-certified critical care physicians, whereas the remaining 178 (59.2%) respondents specialized in other areas, mainly internal medicine, anesthesiology, pulmonary medicine and surgery.

### Sedation practices

Most respondents reported the routine use of a structured sedation scale (281; 92.6%). Almost half of the respondents reported performing daily interruptions of sedation (147; 48.4%), and the same percentage of participants (48.0%) agreed that patients are often over sedated. The existing process of care and current practices are detailed in table 2.

**Table 2:** Attitudes of intensive care unit physicians toward sedation

Written sedation protocol	
Yes	179 (59)
No	124 (41)
Written pain protocol	
Yes	170 (56)
No	133 (44)
Written delirium protocol	
Yes	130 (43)
No	173 (57)
Light sedation is performed	
Yes	158 (52)
No	145 (48)
Daily sedation interruption is performed	
Yes	145 (48)
No	158 (52)

Results expressed as n (%).

**Figure 1:**
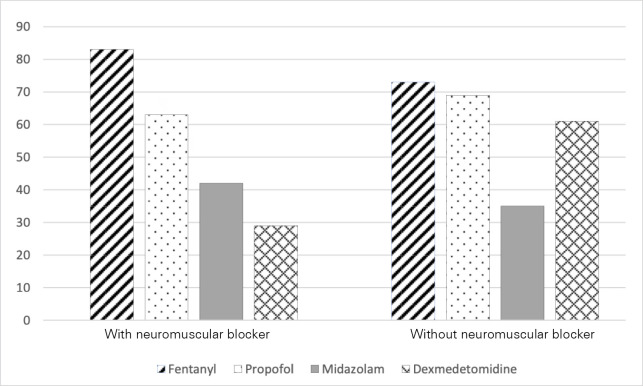
Medications used for sedation management.

Drug regimens for sedation varied widely across respondents (Figure 1), but 34.2% (n = 103) of respondents still used midazolam as their first choice for sedation. Heavy sedation was more common during the COVID-19 pandemic compared to before the pandemic (241; 79.4% *versus* 148; 49.0%; p = 0.01).

We asked physicians for their opinion on 5 strategies to increase adherence to light sedation as per the PADIS guidelines. Most physicians agreed or strongly agreed that a higher (optimally 1:1) nurse-patient ratio (192; 58.2%), the use of a standard sedation scale (180; 54.5%), and written protocols (174; 52.7%) are useful strategies to improve sedation practices. Only 50 (16.7%) respondents reported some difficulty in obtaining access to short-acting medications, such as propofol or dexmedetomidine.

### Comparisons between board-certified critical care physicians and uncertified physicians and between academic and nonacademic institutions in critical care

We performed comparisons between board-certified critical care physicians and physicians without a critical care certification who worked in ICUs. More board-certified critical care physicians had practiced in the ICU for longer than 10 years compared to uncertified physicians (78; 76.5% *versus* 40; 22.0%; p < 0.0001). When compared with uncertified physicians, board-certified critical care physicians more often used sedation scales (87; 85.3% *versus* 91; 50.0%; p < 0.0001) and reported performing more daily interruptions of sedation (46; 45.1% *versus* 46; 25.2%; p = 0.0009). In the ICUs where board-certified critical care physicians worked, sedation targets were more often discussed than in ICUs where uncertified physicians worked (60; 58.8% *versus* 53; 29.1%; p < 0.0001).

We performed the same comparisons between ICU physicians working at academic and nonacademic institutions. No significant differences were observed between physicians from academic institutions *versus* nonacademic institutions.

### Intensive care unit physicians and depth of sedation

During the COVID-19 pandemic, participants reported that patients had a higher chance of receiving midazolam 178; 58.8% *versus* 106; 34.0%; p = 0.05) instead of propofol (131; 43.3% *versus* 212; 70.1%; p = 0.08) or dexmedetomidine (53; 17.5% *versus* 87; 28.9%; p = 0.26).

Participating physicians reported considering with equal emphasis the use of ketamine as an opioid-sparing agent, both in patients with and without COVID-19 (134; 44.3% *versus* 139; 45.9%).

Additionally, participants reported that deep sedation is associated with an increase in length of stay and mortality rate (270; 89.0%); worse functional and cognitive outcomes (289; 95.3%); and significantly increased risk of *delirium* regardless of the type of sedative used (273; 90.1%). The majority of physicians (281; 97.1%) reported that successful provision of light sedation can be performed effectively, irrespective of the type of sedative used, if protocols of targeted and titrated sedative intensity are implemented. However, 31.8% (n = 96) of participants still believed that a light sedation strategy increased the risk of agitation and associated adverse events.

### Economic aspects

Most participants were aware of the costs involved with the use of short-acting drugs, such as propofol (184; 60.8%) or dexmedetomidine (187; 61.8%). However, considering the potential to reduce the time to extubation and the total duration of ICU and hospital stay, participants believed these drugs are cost-effective.

### Propofol for critically ill patients undergoing mechanical ventilation

Only 28.9% (n = 87) of participants reported finding it safe to use propofol routinely for prolonged sedation. In addition, 52.0% (n = 158) of participants found that lipid intake represents a risk even if closely monitored, and 60.6% (n = 184) of participants avoided prolonged use (greater than 7 days). Propofol was reported to be associated with an increased risk of health care-related bloodstream infections among 73.0% (n = 221) of participants.

Despite these significant drawbacks, propofol has achieved widespread acceptance in neurointensive care. Two hundred and sixty-two participants (85.9%) believed that light sedation recommendations could be implemented as long as there was no intracranial hypertension or uncontrolled seizures, and 161 (52.9%) used propofol as the mainstay sedation for the neurocritical care patient.

## DISCUSSION

We conducted a survey of Brazilian ICU physicians aiming to characterize the knowledge and perceived attitudes toward pharmacologic interventions for sedation, including before and during the COVID-19 pandemic.

The 2018 PADIS guidelines suggested that a patient’s current sedation status should be assessed and then frequently reassessed using valid and reliable scales.^(1)^ Before the 2013 Society of Critical Care Medicine (SCCM) guidelines, the surveys demonstrated that less than 50% of physicians reported using sedation protocols,^(4)^ but more recently conducted evaluations showed increasing compliance with those strategies.^(5)^ Currently, the concept of sedation holding has been implemented in most units, and most ICUs have a written sedation guideline.^(6)^

Although most of the studies report self-perception, some audits revealed startling differences between physicians’ statements and actual clinical practice.^(4,5)^ In the current survey, most respondents (92.6%) reported the use of a written sedation protocol. However, the reported frequency of sedation monitoring was clearly insufficient, as most physicians (48.0%) agreed that patients are often over sedated. A partial explanation for this may rely on the fact that 31.8% of participants still believed that a light sedation strategy increased the risk of agitation and associated adverse events. This represents a clear target for educational intervention to change the local culture and clinician behavior.

In 2000, Kress et al. reported that a protocol of daily spontaneous awakening trials reduced the duration of MV and length of stay in intensive care. This study showed that daily spontaneous awakening trials are safe; self-extubation, intensive-care-related complications, myocardial ischemia, and posttraumatric stress disorder did not occur more frequently in patients managed with daily spontaneous awakening trials than in those managed without spontaneous awakening trials.^(7)^ Organizational factors and processes of care are associated with improved outcomes in critically ill patients, such as continuity of care, multidisciplinary rounds, and adoption of protocols. Intensive care unit context factors, such as safety culture, lack of leadership, and lack of interprofessional team support, may play a role as barriers to the effective implementation of PADIS guidelines.^(8)^ In our study, 17% of participants had difficulty accessing fast-acting medications, imposing an important barrier to implementing adequate PADIS guidelines.

Although protocols were previously associated with improved outcomes in critically ill patients,^(9)^ it seems that just having them in the ICU is not enough, since sedation protocols did not decrease the time under MV in some specific settings.^(10)^ Our study suggests that board-certified critical care physicians may have an important role in lighter sedation targets as they more often used sedation scales and reported performing more daily interruptions of sedation than uncertified physicians. Moreover, sedation targets were more often discussed in ICUs with the presence of board-certified critical care physicians, but even then, they were discussed only 58.8% of the time.

Our study suggested that having a board-certified critical care physician on shift was an organizational factor associated with achieving target sedation levels in MV patients. Board-certified physicians may have an important role in lighter sedation targets, as they may be more aware of the importance of light sedation goals than uncertified physicians, such as its possible association with reduced mortality rates. Among 50% of the participants, a reduced staff number was an important barrier to implementing protocols and daily interruption of sedation.

Therefore, the presence of more board-certified critical care physicians in ICUs may ensure that this target will be pursued with more determination. Thus, a high-performance team model can lead to better outcomes.^(8)^ PADIS guidelines also recommend achieving light sedation by daily sedation interruption or targeted sedation. In our survey, only 48.4% of the respondents used daily interruptions in MV patients. Studies have demonstrated that using daily interruption of sedatives is associated with a reduced duration of MV^(11)^ and post-ICU neuropsychologic consequences,^(12)^ as well as improved in-hospital outcomes.^(13)^ Surveyed physicians reported that higher nurse-patient ratios (optimally 1:1) could improve sedation practices and lead to better outcomes.

**Figure 2:**
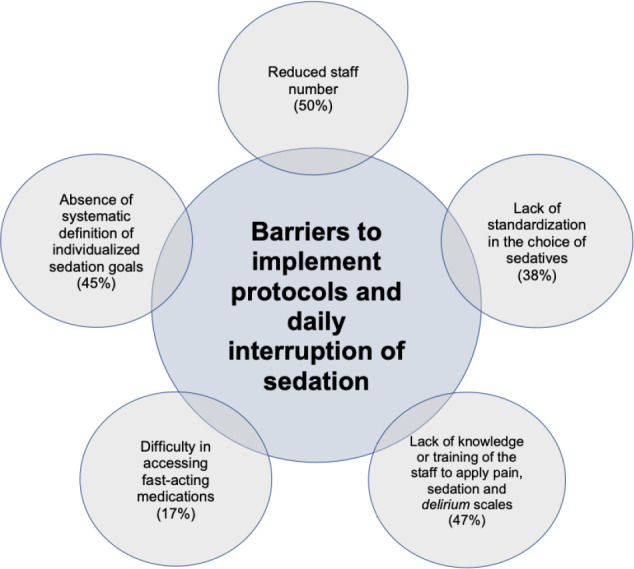
Barriers to implementing protocols and daily interruption of sedation.

There may be several barriers to implementing protocols and daily interruptions of sedation on a regular basis, and they are mostly organizational issues and a feeling of uncertainty regarding the safety of light sedation by assistant physicians (Figure 2). The current PADIS guidelines also recommend that nonbenzodiazepine drugs be used instead of benzodiazepines for the sedation of patients under MV. Garcia et al. performed a meta-analysis comparing the use of propofol with that of midazolam.^(14)^ This study suggests that a propofol-based sedation regimen is cost-effective. These cost savings occur due to the reduced length of ICU stay and the duration of MV. Nonetheless, the use of midazolam remains ingrained in ICUs, as 34.2% of physicians reported prescribing midazolam as the first choice for MV patients.

A multimodal analgesic approach is routinely used to reduce opioid use and optimize analgesia. While opioids remain the mainstay analgesic in critically ill adults, safety concerns associated with their use, particularly sedation, respiratory depression and ileus, are important considerations in some patients.^(15)^ The use of intravenous ketamine is a strategy to reduce opioid use and improve analgesic effectiveness. Participating physicians reported considering the use of ketamine as an opioid-sparing agent, both in patients with and without COVID-19.^(16)^

We observed that many physicians avoid the prolonged use of propofol, since they find that lipid intake represents a risk for hypertriglyceridemia and pancreatitis or health care-related infections, but there is no recommendation to support this practice.^(17)^ This is also an area where the availability of evidence and its dissemination to critical care physicians may help improve adherence to guidelines.

This study highlights that the COVID-19 pandemic has led to changes in some sedation practices. Although changes could have occurred due to the high prevalence of acute respiratory distress syndrome, they still represent a high rate of noncompliance to the PADIS guidelines even for this subgroup of patients. Similar to this survey, a study found a high sedation rate during MV, with midazolam as the most commonly used sedative during the pandemic.^(14)^ We observed a high use of neuromuscular blockers, as well as more frequent use of deep sedation during the pandemic. As the pandemic subsides, it is vital to ensure that these changes in practices are not permanent and focus on the systematic use of evidence-based practices aimed at light sedation and selecting sedatives according to the recommendations of current guidelines.

Regarding the types of sedatives, it is interesting to observe that the most commonly used drugs are a combination of propofol (70.1%) and fentanyl (85.6%), which is quite similar to the North American survey in 2012,^(18)^ the Canadian survey in 2014,^(19)^ the worldwide ABCDEF bundle survey in 2017^(20)^ and the Portuguese survey in 2022.^(21)^ Among 97.1% of physicians, successful provision of light sedation can be performed effectively, irrespective of the type of sedative used, since protocols of targeted and titrated sedative intensity are implemented.

The present survey has some limitations. First, as in any survey, we acknowledge that the possible occurrence of inaccuracies due to poor recollection may result in discrepancies between the self-reported and the actual practice. Second, considering the high numbers of board-certified physicians in the sample, a selection bias may have occurred. However, the sample involved physicians from all geographic regions of the country, including private and public institutions. The majority of participants were from the southeast region, which may have caused sampling bias; however, the concentration of physicians in this region reflects the reality of the distribution of intensive care physicians in our country. Third, although most respondents had more than 5 years of practice, the survey was applied during the COVID pandemic when there was an increased concern about outcomes related to *delirium* and agitation, which may be associated with a recall bias where the systematic error was caused by differences in the accuracy or completeness of the study participants’ recollections regarding events or experiences prior to the pandemic.

## CONCLUSION

This survey provides valuable data on the perceived attitudes of Brazilian intensive care unit physicians regarding sedation before and during the COVID-19 pandemic. Although daily interruption of sedation was a well-known concept and sedation scales were often used by the respondents, insufficient effort was put into frequent monitoring, use of protocols and systematic implementation of sedation strategies.

The difficulties in the care of mechanical ventilation patients during the COVID-19 pandemic have had a negative impact on sedation practices in Brazil. Despite the perception of the benefits linked with light sedation, there is a need to identify improvement targets to propose educational strategies to improve current practices.
