# Supplementary material for: Perceptions and practices regarding light sedation in mechanically ventilated patients: a survey on the attitudes of Brazilian critical care physicians
Source: Rev Bras Ter Intensiva. 2022 Oct-Dec;34(4):426–32. doi: 10.5935/0103-507X.20220278-en (PMC9987013; doi:10.5935/0103-507X.20220278-en)
Supplement: Supplementary file 1 [file rbti-34-04-0426-suppl1.pdf]

Perceptions and practices regarding light sedation in mechanically ventilated patients: A survey on the attitudes of Brazilian critical care physicians

Percepções e práticas sobre sedação superficial em pacientes sob ventilação mecânica: um inquérito sobre as atitudes de médicos intensivistas brasileiros

Vicente Cés de Souza-Dantas[1], Lilian Maria Sobreira Tanaka[2], Rodrigo Bernardo Serafim[3], Jorge Ibrain Figueira Salluh[4]

Questionnaire

1) Do you agree to participate in the study?
( ) Yes. ( ) No.

2) Are you a physician and work in an adult ICU?
( ) Yes. ( ) No.

3) How many years do you have been working in intensive care medicine?
( ) < 5 years. ( ) 5 - 10 years. ( ) > 10 years.

4) Are you a specialist in intensive care medicine?
( ) Yes. ( ) No.

5) What region of Brazil do you work in?
( ) Midwest. ( ) Northeast. ( ) Southeast. ( ) North. ( ) South.

6) What kind of hospital do you work in?
( ) Public. ( ) Private. ( ) University.

7) How many beds does your ICU have?
( ) ≤ 10 beds. ( ) 11 - 20 beds. ( ) > 20 beds.

8) Does your ICU have a residency and/or specialization program in intensive care medicine?
( ) Yes. ( ) No.

9) Does your ICU have a multiprofessional round (at least 5 days a week)?
( ) Yes. ( ) No.

10) Among the protocols below, indicate which are fully implemented (applied to more than 80% of eligible patients) in your ICU (Check all that apply):
( ) Assessment of sedation with validated scales.
( ) Daily interruption of sedation.
( ) Goal-guided sedation.
( ) Assessment of pain with validated scales.
( ) Assessment of *delirium* with validated scales.
( ) Maintenance of light sedation.

11) In your ICU who applies the protocols to patients daily? (Check all that apply):
( ) Nurse. ( ) Nursing technician. ( ) Physiotherapist. ( ) Physician.

12) Regarding the implementation of sedation-guided protocols, what challenges are present in your ICU? (Check all answers that apply):
( ) Reduced number of staff.
( ) Absence of definition of clear sedation goals.
( ) Unfamiliarity or absence of sedation-guided protocols.
( ) Great individual variation (or lack of standardization) in the choice of sedatives to be used.
( ) Difficulty in accessing fast-acting medications (e.g. dexmedetomidine, propofol).
( ) There is no barrier to implementation in the ICU.

13) Considering the degree of importance for the interventions below only for mechanically ventilated patients WITHOUT COVID-19:
- Light sedation should be used from the first day of intubation:
  ( ) Totally agree. ( ) Agree. ( ) Neutral. ( ) Disagree. ( ) Totally disagree.
- Deep sedation should be used until stabilization and later sedation superficial:
  ( ) Totally agree. ( ) Agree. ( ) Neutral. ( ) Disagree. ( ) Totally disagree.
- The preferred sedative is midazolam and can be associated with fentanyl:
  ( ) Totally agree. ( ) Agree. ( ) Neutral. ( ) Disagree. ( ) Totally disagree.
- The preferred sedative is propofol:
  ( ) Totally agree. ( ) Agree. ( ) Neutral. ( ) Disagree. ( ) Totally disagree.
- The preferred sedative is dexmedetomidine:
  ( ) Totally agree. ( ) Agree. ( ) Neutral. ( ) Disagree. ( ) Totally disagree.
- Ketamine can be used, but in exceptional situations:
  ( ) Totally agree. ( ) Agree. ( ) Neutral. ( ) Disagree. ( ) Totally disagree.
- Adjuvant opioids must be used in most patients:
  ( ) Totally agree. ( ) Agree. ( ) Neutral. ( ) Disagree. ( ) Totally disagree.

14) Considering the degree of importance for the interventions below only for mechanically ventilated patients WITH COVID-19:
- Light sedation should be used from the first day of intubation:
  ( ) Totally agree. ( ) Agree. ( ) Neutral. ( ) Disagree. ( ) Totally disagree.
- Deep sedation should be used until stabilization and later sedation superficial:
  ( ) Totally agree. ( ) Agree. ( ) Neutral. ( ) Disagree. ( ) Totally disagree.
- The preferred sedative is midazolam and can be associated with fentanyl:
  ( ) Totally agree. ( ) Agree. ( ) Neutral. ( ) Disagree. ( ) Totally disagree.
- The preferred sedative is propofol:
  ( ) Totally agree. ( ) Agree. ( ) Neutral. ( ) Disagree. ( ) Totally disagree.
- The preferred sedative is dexmedetomidine:
  ( ) Totally agree. ( ) Agree. ( ) Neutral. ( ) Disagree. ( ) Totally disagree.
- Ketamine can be used, but in exceptional situations:
  ( ) Totally agree. ( ) Agree. ( ) Neutral. ( ) Disagree. ( ) Totally disagree.
- Adjuvant opioids must be used in most patients:
  ( ) Totally agree. ( ) Agree. ( ) Neutral. ( ) Disagree. ( ) Totally disagree.

15) Regarding the use of midazolam for mechanically ventilated patients:
( ) Should be considered as a first line sedation.
( ) It is cheaper than propofol.
( ) It is cheaper than dexmedetomidine.
( ) The total cost of hospitalization of a patient sedated with midazolam is less than of patients sedated with propofol or dexmedetomidine.
( ) It is associated with longer duration of mechanical ventilation when compared to other regimes of sedation.

16) Regarding the use of propofol for mechanically ventilated patients:
( ) The use of the 2% formulation is safer.
( ) The costs are not a factor limiting its routine use.
( ) Formulations with EDTA reduce significantly the risk of bloodstream infection.
( ) The lipid supply represents a risk even if monitored.
( ) Prolonged use (> 7 days) represents a significant risk and should be avoided.

17) Regarding patients using neuromuscular blockers:
( ) Benzodiazepines are the sedatives of choice.
( ) Adjuvant opioids must be used in most patients.
( ) Propofol can be used in most patients.
( ) Dexmedetomidine can be used for sedation.

18) Regarding the use of deep sedation in patients under mechanical ventilation:
( ) Deep sedation in first 48 hours of mechanical ventilation no affect the outcome of patients.
( ) Must be implemented systematically in all patients with ARDS.
( ) Has association with increase in duration of hospitalization and mortality.
( ) May be associated with decline in cognitive function even after hospital discharge.
( ) It is associated with the greatest risk of *delirium* in the ICU regardless of the type of sedative used.

19) Regarding the use of light sedation in patients under mechanical ventilation:
( ) It is associated with increased morbidity and mortality from adverse events.
( ) It is associated with a higher risk of long-term events such as post-traumatic stress syndrome.
( ) It can be performed effectively, regardless of the type of sedative used, as long as dose titration protocols are implemented.
( ) It can be achieved with the use of "daily interruption of sedation" or "goal-guided sedation protocol".

20) In neurocritical patients (Check all that apply):
( ) Preferably use midazolam.
( ) Preferably use propofol.
( ) Preferably use other combinations of sedatives (e.g. dexmedetomidine, ketamine).
( ) Superficial sedation recommendations can be implemented as long as there is no in.
